# Primary Study on Effect of Extraction Methods on the Properties and Activities of Polysaccharides from *Geum japonicum var. Chinense F. Bolle*

**DOI:** 10.3390/molecules30010148

**Published:** 2025-01-02

**Authors:** Xuan Chen, Ying-Bo Liu, Yong Deng, Jian-Yong Zhang

**Affiliations:** 1 School of Pharmacy, Zunyi Medical University, Zunyi 563000, China; 18089657392@163.com; 2 Department of Pharmacy, Zunyi Medical And Pharmaceutical College, Zunyi 563006, China; zyyzliuyb@163.com; 3 State Key Laboratory for Quality Ensurance and Sustainable Use of Dao-di Herbs, Beijing 100700, China; 4 Key Laboratory of Basic Pharmacology Ministry Education, Zunyi Medical University, Zunyi 563006, China; 5 Joint International Research Laboratory of Ethnomedicine Ministry of Education, Zunyi Medical University, Zunyi 563000, China

**Keywords:** *Geum japonicum* polysaccharides, extraction method, physicochemical properties, characteristic, antioxidant activity

## Abstract

*Geum japonicum Thunb. var. Chinese F. Bolle*, a traditional Miao medicine with significant clinical potential, is rich in polysaccharides. Despite its importance, there is a scarcity of research on the structure and activities of these polysaccharides. In this study, polysaccharides from *Geum japonicum* (GJPs) were extracted using various methods, including heated reflux extraction (HRE), acidic extraction (ACE), alkaline extraction (AAE), microwave-assisted extraction (MAE), enzymatic extraction (EAE), pressurized liquid extraction (PLE), and deep eutectic solvents extraction (DESE). The extraction yield, physicochemical properties, structural characteristics, and antioxidant activities of these polysaccharides were comprehensively investigated and compared. Physicochemical analysis, including FT-IR spectral features and monosaccharide compositions, revealed that the GJPs are acidic heteropolysaccharides with both *α*- and *β*-configurations. DESE and ACE were the most effective methods for obtaining the highest neutral and acidic sugars with yields of 29.1%/64.2%, and 39.8%/55.6%, respectively. Meanwhile, AAE was preferable for extracting the polysaccharide–protein complex, achieving a yield of 14.21% and exhibiting superior thermal stability. In particular, DESE and PLE showed the best homogeneity with distinct molecular weights of 39.5 kDa and 17.6 kDa, respectively. In addition, biological evaluation indicated that DESE and MAE exhibited relatively stronger antioxidant activities as evidenced by DPPH and ABTS assays. Conversely, ACE demonstrated highest Fe^2+^ chelating ability but the lowest activity in DPPH and ABTS assays. Furthermore, the results of correlation analysis showed that the monosaccharides composition, protein and polyphenol content were significantly associated with the antioxidant activity. The choice of extraction method greatly affects the property and activity of *G. japonicum* polysaccharides. Polysaccharides extracted by deep eutectic solvents from *G. japonicum* show promise as natural antioxidants in the food and medicine industries.

## 1. Introduction

*Geum japonicum Thunb. var. Chinese* F. Bolle, a perennial herb widely distributed in east Asia [1], is valued as a traditional Miao medicine in the Guizhou province of China for its use in treating anemia, nourishing blood, alleviating dizziness, and correcting irregular menstrual irregularities. This herb is known to contain a variety of bioactive compounds, including terpenoids, flavonoids, tannins, and phenylpropanoids, which have been associated with antiviral, anti-inflammatory, cardiovascular, cerebrovascular protection, and lipid-lowering activities [2]. Most of the above-mentioned researchers have primarily focused on small molecules extracted by alcohol or acetone [3], yet the traditional method of utilizing *G. japonicum* involves a water decoction. Our previous study demonstrated that the aqueous extract of *G. japonicum* has a prominent effect on the treatment of blood deficiency [4], with the water extract containing tannins, flavonoids and sugars, notably with a total sugar content reaching up to 41%. As we know, polysaccharides, as macromolecular sugars and key active components in many plant herbs, are known for their excellent biological activities, such as antioxidant, antitumor, and immune regulation [5], suggesting their potential significance in *G. japonicum*. However, research on the extraction, structure, and biological activity of polysaccharides from *G. japonicum* is limited, with most efforts concentrated on optimizing the hot water extraction method [6], which greatly hampered the full exploitation and application of *G. japonicum* sources. Therefore, a great deal of study should be performed on polysaccharides from *G. japonicum*. 

It is well established that the physicochemical and structural properties of polysaccharides are intimately linked to their biological activities. Prior research has indicated that the physicochemical properties and structural characters of polysaccharides are significantly influenced by the extraction methods involved in their preparation process [7]. Extraction is the fundamental procedure for the characterization and exploitation of the active polysaccharides from plant sources, which is critical for determining the compositions and phytochemical properties of natural polysaccharides. Generally, hot water extraction, alkali-assisted extraction, acid-assisted extraction, and advanced extraction techniques such as enzyme-assisted extraction, microwave-assisted extraction, pressurized liquid extraction, and deep eutectic solvent extraction have been used to extract polysaccharides from natural materials. Studies have shown that these various methods result in notable variations in the physicochemical properties and bioactivities of extracted polysaccharide [8,9,10]. For example, Hua Zhu et al. [11] investigated the impact of enzyme-assisted extraction, ultrasound-microwave-assisted extraction, and hot water extraction techniques on the structural characteristics and antioxidant activity of polysaccharides derived from *Dendrobium huoshanense*, finding distinct thermal stability, molecular weight, monosaccharide composition ratio, and antioxidant capacity among the three polysaccharides with enzyme-assisted extraction method displaying broader thermal degradation temperatures and stronger free radical scavenging activities. Similarly, Nour Bhiri et al. [12] used different extraction methods, including hot water maceration, enzyme-assisted extraction, and ultrasonic-assisted extraction, to demonstrate the varying effects on the physicochemical, structural, and functional properties and the biological activities of polysaccharides from *Ononis natrix* leaves. The polysaccharides extracted by water had the highest carbohydrate and uric acid content and exhibited the most potent antioxidant and antibacterial activities. To date, no studied have reported on the yield, physicochemical properties, and functional traits of polysaccharides from *G. japonicum* extracted using different solvent methods. 

The aim of this study is to evaluate the impact of various extraction methods such as heated reflux extraction (HRE), acidic extraction (ACE), alkaline extraction (AAE), microwave-assisted extraction (MAE), enzymatic extraction (EAE), pressurized liquid extraction (PLE), and deep eutectic solvent extraction (DEAE) on the yield and physicochemical properties of *G. japonicum* polysaccharides extracts (GJPs). Thus, the extracts obtained were evaluated by UV-visible absorption spectroscopy, Fourier transform infrared spectroscopy, surface morphological characteristics, thermal stability and in vitro antioxidant activity. Additionally, the correlation of physicochemical properties with antioxidant activities was discussed.

## 2. Results and Discussion

### 2.1. Analysis of the Yield and Physicochemical Property of Crude Polysaccharides

**Figure 1:**
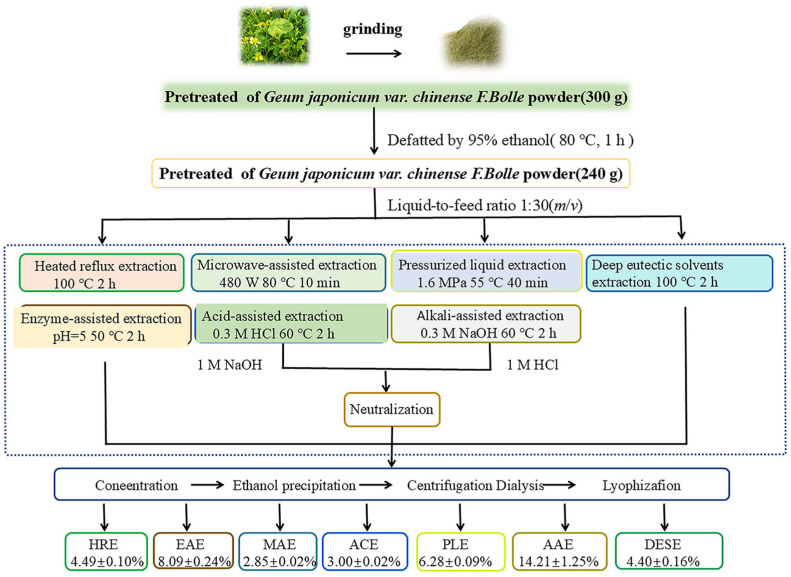
The flowchart of extraction by different methods: heated reflux extraction (HRE), acidic extraction (ACE), alkaline extraction (AAE), microwave-assisted extraction (MAE), enzymatic extraction (EAE), pressurized liquid extraction (PLE), and deep eutectic solvent extraction (DEAE). Data are expressed as the mean of triplicate ± SD.

The extraction techniques employed in this study were primarily based on the methodologies of Tang et al. [13] and Yuan et al. [14] with minor modifications. A visual representation of the extraction is depicted in Figure 1. The physicochemical properties of the crude extract of GJPs prepared by different methods were investigated, including neutral sugar, uronic acid, protein, phenols, and flavonoids. The detailed results are presented in Table 1. Among the extraction methods tested, AAE yielded the highest total polysaccharide content at 14.21 ± 1.25%, which was significantly higher than that obtained using a traditional hot water extraction method, yielding 4.49 ± 0.10%. The order of extraction yields from highest to lowest was AAE > EAE > PLE > HRE > DESE > ACE > MAE, which suggested that the choice of extraction method played an essential role in the yields of the GJPs. The high yield from AAE may be attributed to the acidic solution facilitating cell wall destruction and dissolving polysaccharides. In contrast, microwave showed the lowest yield, which be due to the high power leading to the degradation of polysaccharides [15]. As shown in Table 1, DESE and ACE were particularly effective in extracting the highest total neutral and acidic sugar with percentages of 29.1%/64.2% and 39.8%/55.6%, respectively. The content of neutral sugar, when expressed as glucose equivalents, was highest in ACE (39.82%), followed by EAE (33.73%) and PLE (31.43%), with AAE yielding the lowest at 23.16%. All the obtained GJPs contained amounts of uronic acid, especially ACE, MAE, and DESE exhibiting over 50 percent glucuronide content with the DESE having the highest at 64.24%. This suggests that these samples are all acidic polysaccharides. In addition, all the obtained GJPs contained small amounts of protein and phenols. Notably, only ACE, EAE, and PLE had protein content below 5%, while MAE had the highest polyphenol content at 8.92%. Flavonoids were not detected in any of the GJPs extracted by the methods evaluated in this study. 

#### 2.1.1. UV-Visible Absorption and FT-IR Spectra of Obtained GJPs Extracts

**Figure 2:**
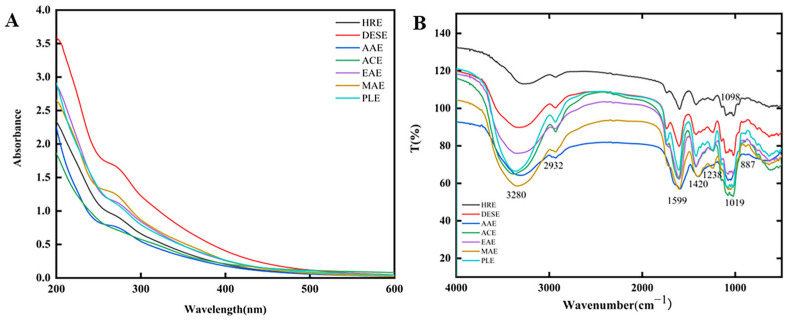
UV-visible absorption (**A**) and FT-IR spectra (**B**) of polysaccharides from different extraction methods. Heated reflux extraction (HRE), acidic extraction (ACE), alkaline extraction (AAE), microwave-assisted extraction (MAE), enzymatic extraction (EAE), pressurized liquid extraction (PLE), and deep eutectic solvent extraction (DESE).

As shown in Figure 2A, with the exception of polysaccharides extracted by ACE and PLE, all other extraction methods yielded polysaccharides that exhibited absorption peaks near 260 nm and 280 nm wavelengths in the UV spectrum, albeit to varying degrees, indicating the presence of small amounts of proteins and nucleic acids, which is consistent with the results of the chemical composition analysis. Fourier-transform infrared (FT-IR) spectroscopy is a valuable tool for identifying the functional groups present in polysaccharides [16]. The FT-IR (between 4000 and 500 cm^−1^) spectra of polysaccharides from *G. japonicum* by different solvents is shown in Figure 2B; no visible differences could be observed among the spectra of different GJPs, indicating that the GJPs extracted by the different methods share similar primary structures. The peaks observed around 3280 and 2932 cm^−1^ corresponded to the stretching vibrations of a hydroxyl group (O-H) and a C-H bond, respectively. These peaks are characteristic of polysaccharides, suggesting that the GJPs possess typical polysaccharide absorption features. Furthermore, an asymmetrical stretching peak at 1420 cm^−1^ and an absorption peak near 1599 cm^−1^ indicate the presence of carbonyl and carboxylic groups (COO-) [17], which is indicative of acidic polysaccharides. The absorption observed between 1000 and 1200 cm^−1^ corresponds to the combined vibrations of C–O and C-C stretching and C-OH bending, indicating the presence of pyranose rings [18,19]. The absorption band at 887 cm^−1^ is attributed to the presence of *β*-type glycosidic linkage, as reported in the literature [20].

#### 2.1.2. Monosaccharide Composition of Obtained GJPs Extracts

**Figure 3:**
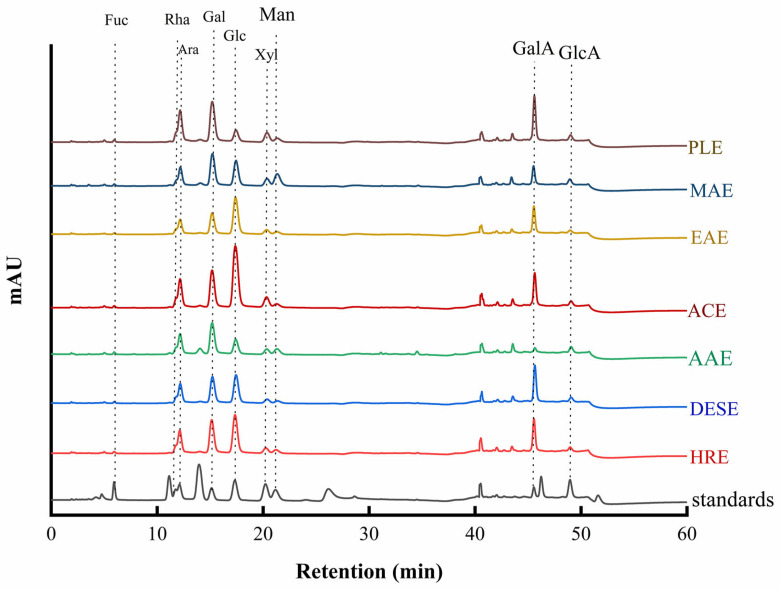
Ion chromatograms of monosaccharide composition. Heated reflux extraction (HRE), acidic extraction (ACE), alkaline extraction (AAE), microwave-assisted extraction (MAE), enzymatic extraction (EAE), pressurized liquid extraction (PLE), deep eutectic solvent extraction (DESE). These abbreviations “Fuc”, “Rha”, “Ara”, “Gal”, “Glc”, “Xyl”, “Man”, “GalA”, and “GlcA” represent fucose, rhamnose, arabinose, galactose, glucose, xylose, mannose, galacturonic acid, and glucuronic acid, respectively.

**Figure 4:**
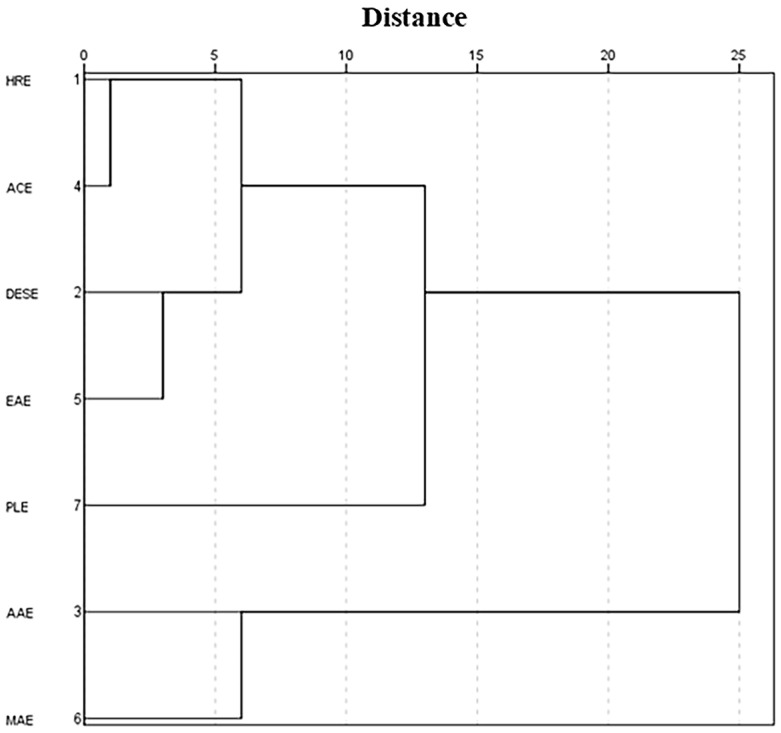
Cluster analysis of the monosaccharide composition of the seven GJPs. Heated reflux extraction (HRE), acidic extraction (ACE), alkaline extraction (AAE), microwave-assisted extraction (MAE), enzymatic extraction (EAE), pressurized liquid extraction (PLE), deep eutectic solvent extraction (DESE).

The compositional analysis of monosaccharides in the GJPs samples was conducted using high-performance anion-exchange chromatography with pulsed amperometric detection (HPAEC-PAD). The monosaccharides present in the hydrolysates of GJPs were identified by comparing their retention times to those of the standards, as depicted in Figure 3. All GJPs samples contained the same type of monosaccharides, which included fucose (Fuc), rhamnose (Rha), arabinose (Ara), galactose (Gal), glucose (Glc), xylose (Xyl), mannose (Man), galacturonic acid (GalA), and glucuronic acid (GlcA), and their proportions are detailed in Table 1. The molar ratios of the monosaccharides varied significantly among the seven polysaccharide samples. To standardize the comparison, the molar ratios were normalized to the content of Gal, which was set as the reference at 1.00. Notably, the molar ratio of Glc was 0.15 in PLE, and in AAE, it was 0.27, which were significantly lower than the ratios observed in ACE and EAE, which were 1.01 and 1.13, respectively. The molar ratio of GalA in AAE was 0.12, which was significantly lower compared to the ratios in HRE, DESE, ACE, EAE, MAE, and PLE, which were 1.07, 1.55, 0.96, 1.48, 0.63, and 1.17, respectively. These findings confirm that all GJPs were acidic heteropolysaccharide, which is in agreement with the results of chemical analysis. To visually assess the similarities and differences in monosaccharide composition across different extraction methods, cluster analysis was conducted, and the results are presented in Figure 4. At a threshold value of 10, the seven extraction methods were categorized into three groups. The first group comprised HRE, ACE, DESE, and EAE, while the second group included PLE, which had the lowest glucose (Glc) content. The third group consisted of AAE and MAE, both of which had lower galacturonic acid (GalA) content. This classification indicates significant variations in monosaccharide composition among the extraction methods with PLE showing the greatest difference from the others. In contrast, methods such as HRE, ACE, DESE, and EAE exhibited more similar monosaccharide compositions. These results underscore the substantial influence of the extraction method on the monosaccharide composition of GJPs. 

#### 2.1.3. Analysis of Molecular Weight

**Figure 5:**
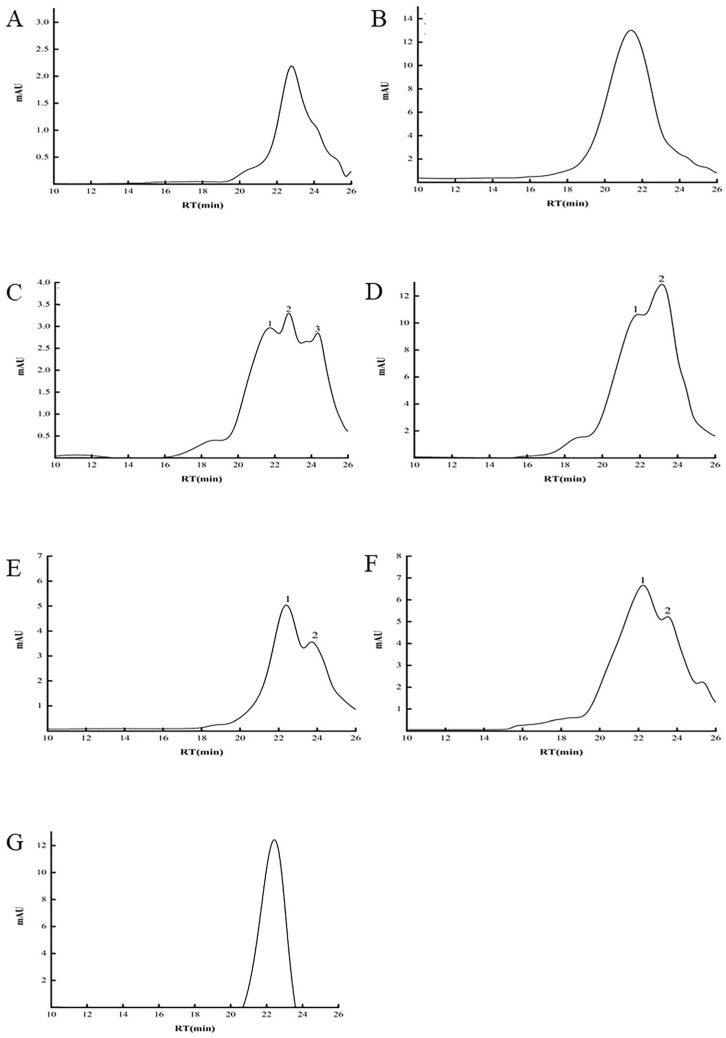
HPSEC-RID chromatograms of GJPs from different extraction methods. (**A**) Heated reflux extraction (HRE); (**B**) deep eutectic solvent extraction (DESE); (**C**) alkaline extraction (AAE); (**D**) acidic extraction (ACE); (**E**) enzymatic extraction (EAE); (**F**) microwave-assisted extraction (MAE); (**G**): pressurized liquid extraction (PLE).

Molecular weight (*M*_w_) is a critical factor influencing the biological activity of polysaccharides. It is observed that some polysaccharides exhibit reduced intrinsic viscosity, enhanced water solubility, and increased biological activity when their molecular weight is lowered [21]. The high-performance size-exclusion chromatography coupled with the refractive index detector (HPSEC-RID) analysis of GJPs is presented in Figure 5 and Table 2, revealing that the molecular weight distributions of polysaccharides vary significantly depending on the extraction method used. Polysaccharides extracted by the traditional hot water method displayed the lowest *M*_w_ at 13.32 kDa. DESE and PLE showed relatively homogenous peaks with higher molecular weights of 30.76 kDa and 17.67 kDa, respectively. Meanwhile, AAE resulted in three main peaks, indicating a complex mixture of polysaccharides with *M*_w_ values of 30.56 kDa, 13.60 kDa, and 3.92 kDa. Furthermore, ACE, EAE, and MAE each showed two main peaks, which indicates the presence of polysaccharides with distinct molecular weights in AAE, ACE, EAE, and MAE. These findings underscore the impact of extraction methods on the molecular weight distribution and heterogeneity of polysaccharide components.

#### 2.1.4. Scanning Electron Microscopy Analysis

**Figure 6:**
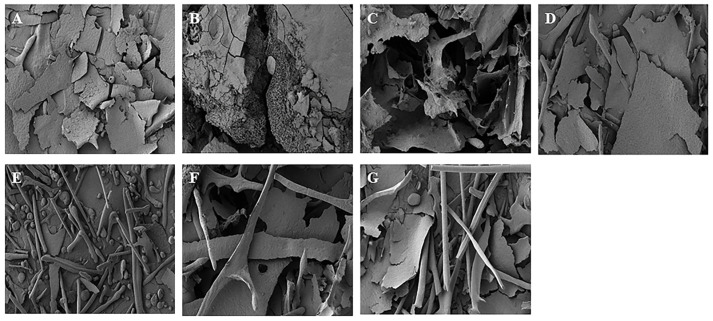
SEM photographs of GJPs from different extraction methods (at 500× magnification). (**A**) Heated reflux extraction (HRE); (**B**) acidic extraction (ACE); (**C**) alkaline extraction (AAE); (**D**) microwave-assisted extraction (MAE); (**E**) enzymatic extraction (EAE); (**F**) pressurized liquid extraction (PLE); (**G**) and deep eutectic solvent extraction (DESE).

The surface structure of polysaccharides is significantly influenced by the extraction, purification, and preparation techniques employed. Scanning electron microscopy (SEM) is an effective technique for examining the surface characteristics of polymer materials at the micron and submicron levels [22]. As shown in Figure 6, the findings indicated that the various extraction methods led to distinct physical alterations in size and shape. The surfaces of HRE and ACE are dominated by lamellar structures with a few rod-like formations at the periphery. In contrast, the EAE and PLE surfaces contain many rod-like structures and have fewer lumps, which may correspond to their lower particle size and relative molecular mass. The DESE results in a compact surface with partially honeycomb-like porosity, possibly related to the higher content of galacturonic acid and proteins, leading to increased particle interaction points and, therefore, a higher degree of aggregation [23]. AAE produces a rough surface with many voids, which is likely a consequence of the corrosive effects in alkaline environments. MAE results in surfaces with irregular, flaky edges, which is probably due to prolonged microwave exposure.

#### 2.1.5. Thermogravimetric Stabilities of Obtained GJPs Extracts

**Figure 7:**
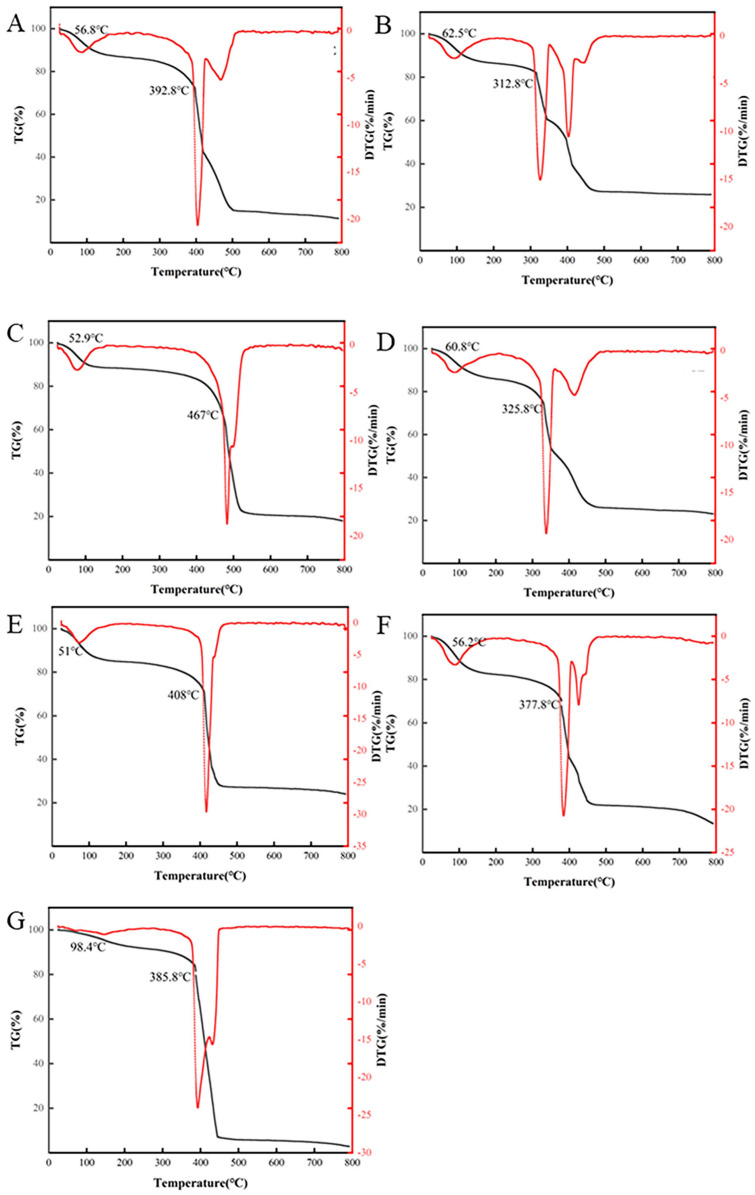
Thermogravimetric analysis (TG) and derivative thermogravimetric analysis (DTG) curves of GJPs. (**A**) Heated reflux extraction (HRE); (**B**) deep eutectic solvent extraction (DESE); (**C**) alkaline extraction (AAE); (**D**) acidic extraction (ACE); (**E**) enzymatic extraction (EAE); (**F**) microwave-assisted extraction (MAE); (**G**) pressurized liquid extraction (PLE).

Thermogravimetric analysis (TGA) is a pivotal technique for assessing the thermal behavior of polysaccharides, providing critical insights into their stability and quality maintenance at specific temperatures and times. This technique is essential for optimizing processing parameters, ensuring product quality, and understanding the physicochemical properties of polysaccharides [24]. Figure 7 presents the TGA results for the GJPs extracted using different methods. The initial phase of mass loss in the TGA curves was observed at temperatures ranging from 50 to 60 °C, which is a phenomenon attributed to the evaporation of moisture from the GJPs. It is noteworthy that the EAE and MAE-derived samples recorded substantially higher mass loss percentages compared to other specimens during this phase of water evaporation. The PLE method registered the lowest mass loss at 3.08%, with the initial mass loss event occurring at a markedly higher temperature of 98.4 °C, indicating variations in water content and hydration patterns among samples extracted by different methods [25]. The subsequent stage of TGA curves is characterized by volatile degradation and depolymerization processes, which involve the breakage and decomposition of chains in polysaccharides, along with a minor degree of polyphenol and protein decomposition. In this stage, the initiation of decomposition temperatures of samples was arranged in descending order as follows: AAE > EAE > HRE > PLE > MAE > ACE > DESE. Notably, the DESE sample exhibited a significantly lower maximal decomposition rate of 15.05%/min compared to the other samples. Furthermore, the mass loss of PLE was considerably more significant (81.69%) than that of the other samples. Those results indicate that the AAE method exhibited the most thermal stable GJPs, while the PLE method yielded the highest amount of volatile compounds. Collectively, these findings underscore the influence of the extraction method on the thermal stability of GJPs [26].

### 2.2. In Vitro Antioxidant Activity Analysis of Obtained GJPs Extracts

#### 2.2.1. DPPH Radical Scavenging Activity

**Figure 8:**
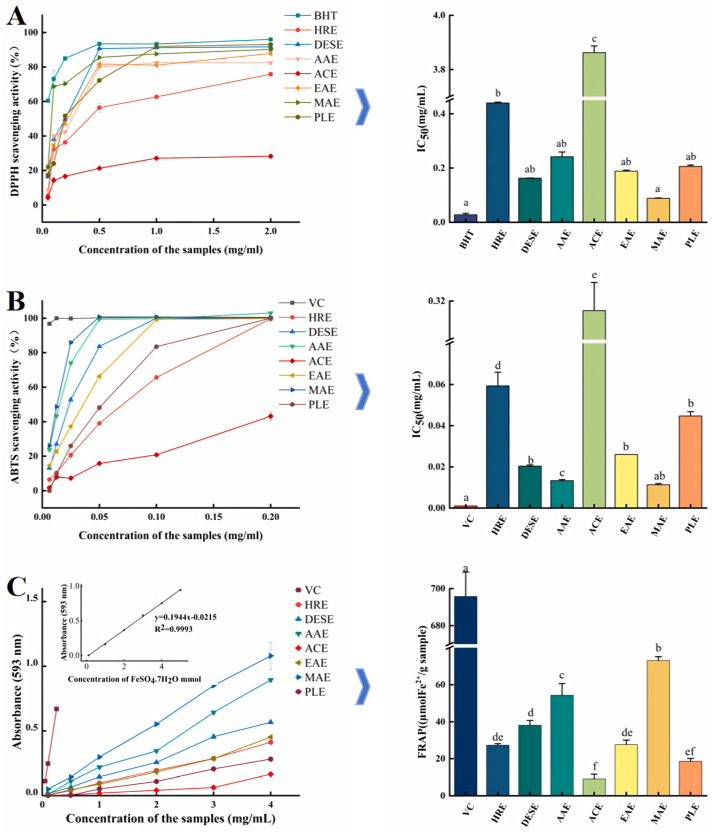
Scavenging activity of GJPs from different extraction methods on DPPH (**A**), ABTS (**B**); and ferric reduction ability (**C**). Heated reflux extraction (HRE), acidic extraction (ACE), alkaline extraction (AAE), microwave-assisted extraction (MAE), enzymatic extraction (EAE), pressurized liquid extraction (PLE), and deep eutectic solvent extraction (DESE). The different superscript letters indicate a significant difference (*p* < 0.05) within the row based on Duncan’s test.

The DPPH radical, as a stabilizing free radical, is often used as an effective tool to measure the free radical scavenging activity of antioxidants [11]. Figure 8A and Table 3 illustrate the dose-dependent DPPH radical scavenging activity of the polysaccharides within the concentration range of 0.01~2.0 mg/mL. The half inhibitory concentration (IC50) values were calculated to compare the DPPH radical scavenging activity among different extraction groups. There was no significant difference in the DPPH radical scavenging activity among the four DESE, EAE, AAE, and PLE groups (*p* > 0.05), suggesting that these groups possessed similar DPPH free radical scavenging ability. Notably, the IC_50_ value of MAE was the lowest at 0.09 mg/mL (*p* < 0.05), indicating superior scavenging abilities compared to the other samples. Conversely, ACE exhibited the largest IC_50_ at 12.25 mg/mL, indicating that the DPPH radical scavenging capacity was lower than that of the other samples.

#### 2.2.2. Scavenging Capacity of ABTS Radical

The antioxidant activity was assessed using the ABTS assay. In this method, catalytic ABTS solutions in the presence of potassium sulfate can undergo electron transfer and generate stable ABTS radicals ABTS^+^ radicals. The antioxidant capacity of the samples is determined by measuring the scavenging capacity of ABTS^+^ radicals. The ABTS radical scavenging ability of the eight GJPs is displayed in Figure 8B and Table 3. As shown in the figure, the ABTS radical scavenging capacity of all GJPs was demonstrated to be concentration-dependent. It was determined that the IC_50_ values of AAE at 0.013 mg/mL and MAE at 0.011 mg/mL were the lowest among all the samples (*p* < 0.05), indicating that these extraction methods yielded polysaccharides with superior scavenging abilities compared to the other methods. Conversely, the IC_50_ of ACE at 0.315 mg/mL was also the largest, suggesting its ABTS’s free radical scavenging ability was inferior to that of the other samples.

#### 2.2.3. FRAP

According to the standard curve of FeSO_4_-7H_2_O, the relationship between the mass concentration of the eight GJPs and the total antioxidant capacity was measured by the FRAP assay. Figure 8C and Table 3 illustrate that these polysaccharides exhibit a certain Fe^3+^ reducing capacity that is concentration-dependent. The FRAP values indicate the ability of the GJPs to reduce Fe^3+^ to Fe^2+^, thereby reflecting their antioxidant potential. It can be seen that MAE produced the highest value of 73.11 μmol Fe^2+^/g (*p* < 0.05), signifying its superior Fe^3+^ reduction capacity compared to the other extraction methods. This was followed by AAE with a value of 54.30 μmol Fe^2+^/g, while ACE showed the lowest value at 9.09 μmol Fe^2+^/g. This result is in agreement with the results of ABTS and DPPH assay, further validating the antioxidant activities of the GJPs. The correlation between the FRAP results and those of ABTS and DPPH suggests a coherent pattern in the antioxidant properties of the GJPs extracted by different methods. The higher FRAP values for MAE and AAE indicate that these extraction techniques may be more effective in preserving or enhancing the antioxidant potential of the polysaccharides. Conversely, the lower FRAP value for ACE suggests that this method may result in polysaccharides with reduced antioxidant activity.

#### 2.2.4. Correlation Analysis

The composition and physicochemical properties of monosaccharides typically influence the antioxidant properties of polysaccharides [27,28]. In order to clarify how the extraction method influences the antioxidant activity of polysaccharides, a correlation analysis was conducted to examine the relationship between the composition and physicochemical properties of monosaccharides and their antioxidant properties. The results are summarized in Table 4. In general, the content of neutral sugars was found to be positively correlated with DPPH and ABTS but negatively correlated with FRAP values. When examining specific monosaccharide components, the content of Ara showed a negative correlation with ABTS and Fe^3+^ clearance (Pearson’s correlation coefficients: r = −0.859, r = −0.927 respectively). Similarly, the content of Rha and GalA was also negatively correlated with DPPH, ABTS, and Fe^3+^ clearance (Pearson’s correlation coefficients: r = −0.683, r = −0.597, r = −0.742, r = −0.079, r = −0.520, r = −0.538, respectively), aligning with previous reports [29,30]. In contrast, the content of Man was positively correlated with the clearance of DPPH, ABTS, and Fe^3+^ (Pearson’s correlation coefficients were r = 0.811, r = 0.822, and r = 0.884, respectively). The content of GlcA showed a positive correlation with ABTS scavenging activities (Pearson’s correlation coefficients of r = 0.845), but the composition of GlcA had no impact on the ability to scavenge DPPH and Fe^3+^ free radicals. Furthermore, the protein and polyphenol content were negatively correlated with DPPH and ABTS activities, while they were positively correlated with FRAP values. This suggests that the protein moiety and covalently linked phenolic components may have diverse roles in the antioxidant properties of GJPs [31]. Based on these results, the antioxidant activity of GJPs is influenced not only by the composition and proportion of monosaccharides and their physicochemical properties but also by the type of free radicals involved. The results of our study are consistent with those of Tang et al. [13].

## 3. Materials and Methods

### 3.1. Materials

Fresh *Geum japonicum* was oven-dried and collected from the local market of Bijie, Guizhou, China. Dextrans with different molecular weights, (2,2′-azino-bis (3-ethylbenzothiazoline-6-sulfonic acid) (ABTS), (1,1-diphenyl-2-picrydrazyl free radical) (DPPH), Cellulase (comes from Trichoderma, 3 U/mg), pectinase (40 U/mg), Papain Papaya (800 U/mg), 2,6-di-tert-butyl-p-cresol (BHT), bovine serum albumin (BSA), and 5×G250 Protein quantitative analysis were bought from Solarbio Science Technology Co., Ltd. (Beijing, China). Monosaccharide standards, D-mannose (Man), L(+)-rhamnose (Rha), D-(+)-galactose (Gal), L-fucose (Fuc), L-(+)-arabinose (Ara), D(+)-glucose (Glc), D-glucuronic acid (GlcA), D-(+)-xylose (Xyl), and galacturonic acid (GalA) were bought from Bo Rui Saccharide Biotech Co., Ltd. (Yangzhou, China). All other reagents used in this study were of analytical grade.

### 3.2. Different Extraction Methods of Obtained GJPs Extracts

#### 3.2.1. Pretreatment of the *Geum japonicum*

The desiccated *Geum japonicum* materials were pulverized and subjected to extraction with 95% ethanol at a liquid-to-solid ratio of 1:20 and 80 °C for 1 h to eliminate pigments, monosaccharides, and other low-molecular-weight impurities. The resulting residues were placed in an Electric Blast Drying Oven (DHG-9123A, Shanghai Jinghong Experimental Equipment Co., Ltd., Shanghai, China) for subsequent drying [32].

#### 3.2.2. Heated Reflux Extraction (HRE)

The powder obtained in Section 3.2.1 was mixed with water at a ratio of 1:30 (g/mL) and extracted at 100 °C in a water bath for 2 h. The extract was centrifuged at 4000 rpm for 10 min using a centrifuge (SN-LSC-40S, Shanghai Shangshu Pu Instruments Equipment Co., Ltd., Shanghai, China). The supernatant was collected and concentrated to 1/4 (*v*/*v*) of its initial volume; then, it was mixed with 95% ethanol at a ratio of 1:4 (*v*/*v*) and stored at 4 °C overnight. Afterwards, the precipitate was collected, redissolved, deproteinized using the Sevag method, and dialyzed (molecular weight cut-off 3000 Da) for 72 h. The resulting polysaccharide was obtained using a freeze-drier (1-16R, Hunan Kecheng Instruments Equipment Co., Ltd., Changsha, China) and designated as HRE. 

#### 3.2.3. Acid-Assisted Extraction (ACE) 

The powder from Section 3.2.1 was mixed with a 0.3 M HCl solution at a ratio of 1:30 (g/mL) and heated in a water bath at 60 °C for 2 h. After centrifugation, the supernatant was neutralized with a 1 M alkaline solution. The subsequent procedures were identical to those for HRE, and the product was named as ACE.

#### 3.2.4. Alkali-Assisted Extraction (AAE) 

The powder from Section 3.2.1 was mixed with a 0.3 M NaOH solution at a ratio of 1:30 (g/mL) and heated in a water bath at 60 °C for 2 h. After centrifugation, the supernatant was neutralized with a 1 M acid solution. The subsequent procedures were similar to those for HRE, and the product was designated as AAE.

#### 3.2.5. Enzyme-Assisted Extraction (EAE)

Eight grams of powder from Section 3.2.1 was mixed with 240 mL of 0.05 M disodium hydrogen phosphate–citric acid buffer (pH = 5) containing cellulase (40 mg), pectinase (40 mg), and papain (40 mg from Papaya). The mixture was incubated at 50 °C for 2 h and then heated to inactivate the enzymes (90 °C, 20 min). The subsequent procedures were similar to those for HRE, and the product was named as EAE.

#### 3.2.6. Microwave-Assisted Extraction (MAE)

The powder from Section 3.2.1 was added to distilled water at a ratio of 1:30 (g/mL) and extracted using a microwave apparatus (MK]-J1-3, Qingdao Microwave Applied Technology Co., Ltd., Qingdao, China) at 80 °C for 10 min with 480 W of microwave power. The subsequent procedures were similar to those for HRE, and the product was named as MAE.

#### 3.2.7. Pressurized Liquid Extraction (PLE)

Eight grams of powder from Section 3.2.1 was extracted with 240 mL of distilled water using a high-pressure reactor (LEC-300, Shanghai Laibei Scientific Instruments Co., Ltd., Shanghai, China) at 55 °C and 1.6 MPa for 40 min. The subsequent procedures were similar to those for HRE, and the product was named as PLE.

#### 3.2.8. Deep Eutectic Solvents Extraction (DESE)

Eight grams of powder from Section 3.2.1 was mixed with 240 mL of deep eutectic solvent (choline chloride-ethylene glycol, 1:3), heated at 80 °C to clarify the liquid, and then mixed with 40% deionized water. The extraction was performed at 100 °C for 2 h. The subsequent procedures were similar to those for HRE, and the product was named as DESE.

### 3.3. Comparison of Physicochemical Property of Crude Polysaccharides

The content of neutral polysaccharide was quantified using the phenol–sulfuric acid method, employing glucose (Glc) serving as the calibration standard at an absorbance wavelength of 490 nm [33]. To measure the uronic acid content, the *m*-Hydroxybiphenyl colorimetric method was performed, utilizing galacturonic acid (GalA) as the reference standard at 314 nm [34]. The protein content was assessed by the Coomassie Brilliant Blue G-250 assay with bovine serum albumin (BSA) as a reference standard at 595 nm [35]. The determination of phenolic compounds was achieved via the Folin–Ciocalteu colorimetric method with gallic acid as the calibration standard at 765 nm [36]. Additionally, a calibration curve for flavonoid quantification was established using the sodium nitrite–aluminum nitrate–sodium hydroxide colorimetry method, with rutin as the standard, and absorbance was recorded at 510 nm [37].

### 3.4. Comparison of Structure of Polysaccharides

#### 3.4.1. Determination of UV-Vis and IR Spectra

Polysaccharide samples were prepared at a concentration of 1 mg/mL in deionized water for ultraviolet (UV)-visible spectroscopy. Absorption spectra were recorded using a UV spectrophotometer (N_4_S, Shanghai Yidian Scientific Instrument Co., Shanghai, China) over the 200–600 nm. For infrared spectroscopy, polysaccharide samples were pelletized with KBr, and the IR spectra were recorded in the wavenumber range of 4000–500 cm^−1^ using a Fourier-transform infrared spectrometer (Nicolet IS20, Thermo Scientific, Waltham, MA, USA).

#### 3.4.2. Determination of Monosaccharide Compositions

The monosaccharides composition of polysaccharides was measured by high-performance anion-exchange chromatography coupled with a pulsed amperometric detector (HPAEC-PAD) on a Dionex ICS 5000 system (Thermo Scientific, Waltham, MA, USA) [38]. Briefly, samples at a concentration of 5.0 mg/mL were hydrolyzed with 3.0 M trifluoroacetic acid (2.0 mL) at 120 °C for 3 h. Post-hydrolysis, the samples were transferred to tubes, dried under a stream of nitrogen, and washed with methanol three times. Finally, the residues were redissolved in 5 mL H_2_O, diluted tenfold, and centrifuged at 12,000 rpm for 5 min. The supernatant was chromatographed on a Carbopac^TM^ PA20 (3 × 150 mm, 10 μm). The injection volume was 25 μL with an eluent flow rate of 0.3 mL/min at 30 °C. The mobile phases consisted of H_2_O (A), 250 mM NaOH (B), and 500 mM NaOH and 50 mM NaAc (C). The elution gradient was as follows: 0 min A/B/C (98:2:0, *v*/*v*/*v*), 23 min A/B/C (98:2:0, *v*/*v*/*v)*, 23.1 min A/B/C (80:20:0, *v*/*v*/*v*), 33 min A/B/C (80:20:0, *v*/*v*/*v*), 33.1 min A/B/C (80:0:20, *v*/*v*/*v*), 46 min A/B/C (80:0:20, *v*/*v*/*v*), 46.1 min A/B/C (20:0:80, *v*/*v*/*v*), 66 min A/B/C (20:0:80, *v*/*v*/*v*), 66.1 min A/B/C (98:2:0, *v*/*v*/*v*), 80 min A/B/C (98:2:0, *v*/*v*/*v*). Fifteen monosaccharide standards were applied to establish the calibration curves, and data acquisition and processing were performed using Chromeleon 7.2 software (ThermoFisher Scientific, Waltham, MA, USA).

#### 3.4.3. Determination of Molecular Weight

The molecular weights of polysaccharides were conducted using high-performance size-exclusion chromatography with refractive index detection (HPSEC-RID) [39]. Samples were dissolved in 0.05 M NaCl at a concentration of 3 mg/mL, filtered through a 0.45 μm membrane, and analyzed on an Agilent 1200 system (Agilent Technologies, Santa Clara, CA, USA) equipped with two columns in series (Biomac-SEC-WR 5 μm, 8.0 × 300 mm, Bo Rui Saccharide Biotech Co., Ltd., Yangzhou, China). The injection volume was 50 μL, and the eluent was a 0.05 mol/L NaCl solution at a flow rate of 0.6 mL/min at 30 °C. The calibration curve was obtained from dextran with different molecular weights (10, 20, 30, 40, 60, and 200 kDa).

#### 3.4.4. Scanning Electron Microscopy (SEM)

Microstructural examination was performed using scanning electron microscopy (Merlin, Zeiss LTD., Oberkochen, Germany) at an accelerating voltage of 5.0 kV. Samples were mounted on slides using double-sided adhesive tape and sputter-coated with gold powder prior to imaging.

#### 3.4.5. Thermal Analysis

Thermogravimetric analysis (TGA) and derived-thermogravimetric analysis (DTG) were conducted to observe the thermal properties of the polysaccharides using a thermal analyzer (TGA2, METTLER TOLEDO, Zurich, Switzerland). The analyses were conducted over a temperature range of 30–800 °C at a heating rate of 10 °C/min. Approximately 5 mg of each sample was used with an empty aluminum pan as the reference.

### 3.5. In Vitro Antioxidant Activity Evaluation of Polysaccharides

#### 3.5.1. DPPH+ Radical Scavenging Activity

The capacity to scavenge DPPH^+^ free radicals was evaluated according to protocols described in previous research [40] with butylated hydroxytoluene (BHT) serving as a positive control. A 0.2 mM solution of DPPH was prepared in ethanol and stored in the dark. Various concentrations of the polysaccharide solutions (0.05, 0.1, 0.2, 0.5, 1.0, 2.0 mg/mL, 1.0 mL each) were mixed with 1.0 mL of the DPPH–ethanol solution. These mixtures were then incubated at room temperature in the dark for 30 min, after which the absorbance was measured at 517 nm against a blank of anhydrous ethanol. The DPPH^+^ radical scavenging activity was calculated using the following equation:

(1) $$\mathrm{Scavenging}\;\mathrm{activity}\;(\%)=(1-\frac{A_{1}-A_{2}}{A_{0}})\;\times \;100\%$$

where A_0_ was the absorbance of the control group (replacing the sample solution with water), A_1_ was the absorbance of the test group, and A_2_ is the absorbance of the sample with anhydrous ethanol instead of DPPH–ethanol solution.

#### 3.5.2. Assay of ABTS+ Scavenging Activity

The ABTS^+^ radical scavenging activity of the polysaccharides was evaluated employing a method previously reported [41]. An equivalent volume of K_2_S_2_O_8_ (2.45 mmol/L) was added to an aqueous solution of ABTS (7 mmol/L), and the ABTS^+^ solutions were generated by incubating the mixture in the dark for 16 h at ambient temperature. The radical-containing solutions were then adjusted with phosphate-buffered saline (PBS, pH 7.4) to achieve an absorbance of 0.70 ± 0.02 at 734 nm. Subsequently, 2.0 mL of polysaccharide solutions with varying concentrations (ranging from 6.25 to 200 μg/mL) was mixed with 2 mL of the diluted ABTS solution. The absorbance of these mixtures at 734 nm was recorded after a 5-min reaction period in the dark. Ascorbic acid (Vc) served as a positive control. The ABTS scavenging activity was calculated using the following:

(2) $$\mathrm{Scavenging}\;\mathrm{activity}\;(\%)=(1-\frac{A_{1}-A_{2}}{A_{0}})\;\times \;100\%$$

where A_0_ was the absorbance of the control group (water instead of the sample solution), A_1_ was the absorbance of the test group, and A_2_ was the absorbance of the sample with phosphate buffer instead of ABTS.

#### 3.5.3. FRAP Assay

The FRAP assay was conducted based on a previously published protocol with certain modifications [42]. The FRAP reagent was prepared by mixing 0.3 M acetate buffer (pH 3.6) with a 10 mmol/L 2,4,6-tripyridyl-s-triazine (TPTZ), 0.04 M hydrochloric acid solution, and 0.04 M FeCl_3_ in a 10:1:1 ratio. A volume of 20 μL of the polysaccharide sample solutions, each at varying concentrations (0.1, 0.5, 1.0, 2.0, 3.0, 4.0 mg/mL), as added into 1.8 mL of FRAP reagent and thoroughly mixed. The resulting mixtures were incubated at 37 °C in the dark for 10 min, following which the absorbance was measured at 593 nm. A calibration curve was generated using a series of FeSO_4_ standard solutions with concentrations ranging from 100 to 5000 µM. The results were expressed as the FeSO_4_ equivalents of antioxidant capacity (µM FeSO_4_·7H_2_O/g).

### 3.6. Statistical Analysis

All experimental results were repeated at least three times and expressed as mean ± standard error. Statistically significant difference results were obtained by one-way ANOVA and Duncan’s test (*p* < 0.05). Correlation analyses were estimated using Pearson’s correlation coefficient in SPSS 29.0 software (IBM, Chicago, IL, USA). Graphical processing was performed using Origin 2019 software (Origin Lab, Northampton, MA, USA).

## 4. Conclusions

This study has demonstrated that the choice of extraction method significantly influences the yield, properties, structure, and bioactivity of polysaccharides extracted from *G. japonicum*. The analysis of seven polysaccharides sample revealed disparities in molecular weights, morphologies, and UV spectra, yet they showed similar FT-IR spectra and monosaccharide compositions, albeit with varying molar ratios. Both the AAE and EAE techniques were found to be highly efficient in terms of extraction yields. However, the relatively low sugar content in these extracts intimates the potential coextraction of non-sugar components, which may adversely affect the purity of polysaccharides. Conversely, the ACE was more effective in enhancing sugar content and reducing protein and polyphenol impurities, thereby yielding more purified polysaccharide extracts. However, ACE had a lower extraction efficiency, and the antioxidant activity of the resulting extracts was found to be suboptimal. Similarly, the MAE technique also showed unsatisfactory extraction efficiency, and the antioxidant capacity of PLE extracts was inferior to that of DESE extracts. Under comprehensive evaluation, DESE emerged as a superior method, yielding higher polysaccharide content with lower protein and phenol levels, and exhibiting more homogeneous molecular weight distributions compared to the other methods. Moreover, DESE presented relatively higher antioxidant properties. In addition, DESE is an innovative, environmentally friendly extraction technique that offers better biodegradability, adjustable viscosity, and reduced toxicity, making it a promising approach for polysaccharide extraction with environmental and economic benefits [43]. Hence, the use of deep eutectic solvents for extracting polysaccharides from *G. japonicum* is deemed a more advantageous method. Future studies will focus on the detailed structural characterization and exploration of the structure–activity relationships of purified *G. japonicum* polysaccharide fractions to further elucidate their potential applications in traditional Chinese medicine and other therapeutic areas.

## Figures and Tables

**Table 1:** Extraction yields and chemical compositions of GJPs by different extraction methods.

Item	HRE	DESE	AAE	ACE	EAE	MAE	PLE
Yield (%)	4.49 ± 0.10 ^d^	4.40 ± 0.16 ^d^	14.21 ± 1.25 ^a^	3.00 ± 0.02 ^e^	8.09 ± 0.24 ^b^	2.85 ± 0.02 ^e^	6.28 ± 0.09 ^c^
Neutral sugar (%)	27.17 ± 1.2 ^e^	29.13 ± 0.54 ^d^	23.16 ± 0.02 ^f^	39.82 ± 0.37 ^a^	33.73 ± 0.71 ^b^	29.40 ± 0.37 ^d^	31.43 ± 46 ^c^
Uronic acid (%)	37.72 ± 0.98 ^d^	64.24 ± 0.86 ^a^	13.81 ± 0.88 ^f^	55.62 ± 2.9 ^b^	20.57 ± 0.73 ^e^	51.34 ± 1.16 ^c^	49.40 ± 0.46 ^c^
Protein (%)	5.94 ± 0.03 ^b^	5.56 ± 0.06 ^c^	7.87 ± 0.08 ^a^	1.65 ± 0.05 ^f^	3.25 ± 0.08 ^d^	8.09 ± 0.24 ^a^	2.22 ± 0.17 ^e^
Phenols (%)	3.12 ± 0.02 ^e^	4.68 ± 0.04 ^c^	7.97 ± 0.13 ^b^	1.01 ± 0.08 ^f^	3.57 ± 0.01 ^d^	8.92 ± 0.14 ^a^	3.00 ± 0.03 ^e^
Flavonoids	/	/	/	/	/	/	/
Main monosaccharide composition (molar ratio, %)							
Fuc	0.04	0.04	0.05	0.04	0.04	0.04	0.03
Rha	0.19	0.20	0.20	0.21	0.22	0.16	0.20
Ara	0.43	0.46	0.40	0.48	0.44	0.36	0.48
Gal	1.00	1.00	1.00	1.00	1.00	1.00	1.00
Glc	0.70	0.65	0.27	1.01	1.13	0.46	0.15
Xyl	0.13	0.12	0.14	0.22	0.17	0.18	0.18
Man	0.10	0.09	0.19	0.09	0.12	0.39	0.09
GalA	1.07	1.55	0.12	0.96	1.48	0.63	1.17
GlcA	0.07	0.09	0.11	0.07	0.08	0.09	0.07

The values represent the mean ± standard deviation (*n* = 3). The different superscript letters indicate a significant difference (*p* < 0.05) within the row based on Duncan’s test. These abbreviations “Fuc”, “Rha”, “Ara”, “Gal”, “Glc”, “Xyl”, “Man”, “GalA”, and “GlcA” represent fucose, rhamnose, arabinose, galactose, glucose, xylose, mannose, galacturonic acid, and glucuronic acid, respectively. Heated reflux extraction (HRE), acidic extraction (ACE), alkaline extraction (AAE), microwave-assisted extraction (MAE), enzymatic extraction (EAE), pressurized liquid extraction (PLE), and deep eutectic solvent extraction (DESE). Data are expressed as the mean of triplicate ± SD. The symbol “/” indicates that the value was not detected.

**Table 2:** Molecular weight distributions of GJPs.

Methods	RT (min)	Mw (kDa)
HRE	22.79	13.32
DESE	21.40	39.54
AAE	21.72 ① 22.76 ② 24.35 ③	30.76 ① 13.6 ② 3.92 ③
ACE	21.92 ① 23.17 ②	26.29 ① 9.84 ②
EAE	22.39 ① 23.72 ②	18.23 ① 6.41 ②
MAE	22.23 ① 23.51 ②	20.67 ① 7.57 ②
PLE	22.43	17.67

Symbols ①, ②, and ③ represent 1, 2, and 3 in Figure 5, respectively. heated reflux extraction (HRE), acidic extraction (ACE), alkaline extraction (AAE), microwave-assisted extraction (MAE), enzymatic extraction (EAE), pressurized liquid extraction (PLE), and deep eutectic solvent extraction (DESE).

**Table 3:** IC_50_ and FRAP values of free radical scavenging of GJPs extracted by different methods.

Free Radical	Control/Sample IC_50_ (mg/mL)							
	BHT/VC	HRE	DESE	AAE	ACE	EAE	MAE	PLE
DPPH	0.031 ± 0 ^a^	0.425 ± 0 ^b^	0.162 ± 0 ^ab^	0.212 ± 0.01 ^ab^	16.670 ± 0.02 ^c^	0.187 ± 0 ^ab^	0.089 ± 0 ^a^	0.208 ± 0 ^ab^
ABTS	0.004 ± 0 ^a^	0.060 ± 0 ^d^	0.022 ± 0 ^b^	0.013 ± 0 ^c^	0.287 ± 0.01 ^e^	0.029 ± 0 ^b^	0.116 ± 0 ^ab^	0.047 ± 0 ^b^
Fe2+	695.730 ± 13.07 ^a^	27.302 ± 0.81 ^de^	38.133 ± 2.57 ^d^	54.304 ± 6.28 ^c^	9.096 ± 2.54 ^f^	27.624 ± 2.46 ^de^	73.115 ± 2.05 ^b^	18.574 ± 1.55 ^ef^

Heated reflux extraction (HRE), acidic extraction (ACE), alkaline extraction (AAE), microwave-assisted extraction (MAE), enzymatic extraction (EAE), pressurized liquid extraction (PLE), deep eutectic solvent extraction (DEAE). 2,2-Diphenyl-1-picrylhydrazyl (DPPH), 2,2′-azino-bis (3-ethylbenzothiazoline-6-sulfonic acid) (ABTS). The values represent the mean ± standard deviation (*n* = 3). The different superscript letters indicate a significant difference (*p* < 0.05) within the row based on Duncan’s test.

**Table 4:** Matrix for correlation analysis (*p* < 0.05).

	Fuc	Rha	Ara	Gal	Glc	Xyl	Man	GalA	GlcA	Protein	Phenols	Neutral Sugar	Uronic Acid
DPPH	−0.059	−0.683	−0.678	1.410	−0.334	−1.430	0.811 *	−0.079	0.410	−0.556 **	−0.585 **	0.756 **	0.299
ABTS	0.494	−0.597	−0.859 *	0.560	−0.422	−0.314	0.822 *	−0.520	0.845 *	−0.611 **	−0.659 **	0.990 **	0.331
Fe^3+^	0.470	−0.742	−0.927 **	0.536	−0.409	−0.322	0.884 **	−0.538	0.753	0.905 **	0.974 **	−0.644 **	−0.195

* Notes the correlation was significant at 0.05 level (two tailed), while ** indicates the correlation was significant at the level of 0.01 (two tailed). These abbreviations “Fuc”, “Rha”, “Ara”, “Gal”, “Glc”, “Xyl”, “Man”, “GalA”, and “GlcA” represent fucose, rhamnose, arabinose, galactose, glucose, xylose, mannose, galacturonic acid, and glucuronic acid, respectively. 2,2-Diphenyl-1-picrylhydrazyl (DPPH), 2,2′-azino-bis (3-ethylbenzothiazoline-6-sulfonic acid) (ABTS).

## Data Availability

The data presented in this study are available on request from the corresponding author due to restriction.
